# Effect of Leaf Type and Pesticide Exposure on Abundance of Bacterial Taxa in Mosquito Larval Habitats

**DOI:** 10.1371/journal.pone.0071812

**Published:** 2013-08-05

**Authors:** Ephantus J. Muturi, Benedict O. Orindi, Chang-Hyun Kim

**Affiliations:** 1 Illinois Natural History Survey, University of Illinois at Urbana-Champaign, Illinois, United States of America; 2 Biostatistics Unit, International Centre of Insect Physiology and Ecology, Nairobi, Kenya; Leiden University, The Netherlands

## Abstract

Lentic freshwater systems including those inhabited by aquatic stages of mosquitoes derive most of their carbon inputs from terrestrial organic matter mainly leaf litter. The leaf litter is colonized by microbial communities that provide the resource base for mosquito larvae. While the microbial biomass associated with different leaf species in container aquatic habitats is well documented, the taxonomic composition of these microbes and their response to common environmental stressors is poorly understood. We used indoor aquatic microcosms to determine the abundances of major taxonomic groups of bacteria in leaf litters from seven plant species and their responses to low concentrations of four pesticides with different modes of action on the target organisms; permethrin, malathion, atrazine and glyphosate. We tested the hypotheses that leaf species support different quantities of major taxonomic groups of bacteria and that exposure to pesticides at environmentally relevant concentrations alters bacterial abundance and community structure in mosquito larval habitats. We found support for both hypotheses suggesting that leaf litter identity and chemical contamination may alter the quality and quantity of mosquito food base (microbial communities) in larval habitats. The effect of pesticides on microbial communities varied significantly among leaf types, suggesting that the impact of pesticides on natural microbial communities may be highly complex and difficult to predict. Collectively, these findings demonstrate the potential for detritus composition within mosquito larval habitats and exposure to pesticides to influence the quality of mosquito larval habitats.

## Introduction

Mosquitoes are important vectors of diseases such as malaria, dengue, Rift Valley fever, and yellow fever which collectively are responsible for ≈98% of the total burden due to vector-borne diseases [1], . These vectors develop in a variety of natural and artificial lentic environments such as ground pools, tree holes, discarded cans, waste tires, and storm water catch basins. The bulk of carbon inputs for these habitats is derived from terrestrial organic matter, mainly leaf litter, with occasional pulses of animal detritus and nutrients from agricultural runoffs and rain water [3], [4]. The detritus is colonized by microbial communities (e.g. bacteria, fungi) which aid in litter decomposition, facilitating its entry into food webs. The breakdown of detritus by microbes produces chemical cues that act as oviposition attractants and stimulants to gravid mosquitoes [5]–[7]. In addition, the microbial assemblages associated with detritus provide a critical food resource for mosquito larvae [8], [9]. Therefore, microbial communities in mosquito larval habitats may influence mosquito population dynamics and spatial distribution.

Previous studies have shown that leaves from different plant species support variable amounts of microbial growth and differentially affect mosquito performance and the outcome of interspecific competition [10]–[12]. However, little is known about the community structure of microbial communities from different species of leaves and their impact on mosquitoes. Moreover, although these microbes are sensitive to changes in environmental conditions, it is unknown how they respond to common natural and anthropogenic environmental stressors in larval habitats.

The objectives of this study were to 1) determine the abundance of major taxonomic groups of bacteria in different types of leaf litter and to 2) investigate potential changes in bacterial community structure and abundance in response to environmentally-relevant concentrations of common agricultural pesticides with different modes of action on the target organisms including permethrin (pyrethroid insecticide), malathion (organophosphate insecticide), glyphosate and atrazine (herbicides). Agricultural pesticides are anthropogenic stressors that are deliberately introduced into the environment over large spatial scales. These chemicals may unintentionally contaminate aquatic systems including those occupied by mosquito larvae through spray-drift, leaching or surface runoffs, and are currently among the leading sources of ground and surface water pollution [13]–[15]. Acute effects of pesticides on mosquitoes and other non-target organisms are often easily detected in toxicity tests during assessment of the pesticide. In contrast, the chronic effects of pesticides usually promoted by exposure to low pesticide concentrations are difficult to detect because they are not immediately apparent and may operate through the food chain. Pesticides are known to alter the community function and structure of microbial communities in freshwater ecosystems [16]–[18] and their presence in mosquito larval habitats may therefore alter the quality and quantity of food for mosquito larvae depending on how they affect the bacterial taxa that contribute to mosquito growth and survival. In fact, there are cases where application of insecticides in the larval habitats result in larger and more fecund females even when there are no apparent differences in survival rates between pesticide treatments and controls [19]. This could be due to pesticide-induced changes in microbial abundance and community structure.

We therefore tested the hypotheses that 1) leaf species support different quantities of major taxonomic groups of bacteria and that 2) exposure to pesticides at environmentally relevant concentrations can alter bacterial abundance and community structure in mosquito aquatic habitats.

## Materials and Methods

### Collection of leaf litter

Senescent leaves of 7 plant species were collected for use in this study. These included grass (*Setaria* sp.), sugar maple (*Acer saccharum*), hackberry (*Celtis occidentalis*), bush honeysuckle (*Lonicera* spp), northern red oak (*Quercus rubra*), eastern white pine (*Pinus strobus*) and live oak (*Quercus virginiana*). Leaves of all plant species except live oak were collected at the University of Illinois, Trelease Woods and South Farm woodlots with permission from the manager of the University of Illinois natural areas. Live oak leaves were collected in Dr. Barry Alto's private property in Vero Beach, Florida with his permission. The litter of each species was pooled, mixed, air-dried, and stored in litter bags at room temperature. None of these plants is listed as an endangered or protected species.

### Experimental design

Experimental microcosms (400 ml plastic beakers) consisted of 35 detritus × pesticide treatments replicated four times (140 containers). The microcosms were established by adding 1 g of senescent leaves of one of 7 plant species listed above in 350 ml of de-ionized water. These plants were chosen based on their rate of decay (slow versus rapid decay), their dominance in central Illinois (except live oak), and their significant contribution of leaf litter to container habitats occupied by mosquito larvae such as tires, tree holes, and catch basins (Muturi, personal observations). Leaves of grass, sugar maple, hackberry, and bush honeysuckle decompose rapidly while those of eastern white pine, northern red oak, and live oak decompose slowly [10], [20], [21]. On day 7, the microcosms were treated with 35 µl of acetone or an equal volume of 10,000 mg/L of one of four agricultural pesticides that are commonly detected in fresh water ecosystems; permethrin (pyrethroid), malathion (organophosphate), glyphosate (glycine phosphonate herbicide) and atrazine (triazine herbicide) in acetone [13], [14]. This resulted in a final concentration of 0 or 1 mg/L for each pesticide.

Two days after adding the pesticides, 1 ml aliquots of water samples were taken from each container and stored at −80°C until further processing. The water samples were later centrifuged at 12000×g for 10 minutes and the resulting pellet was re-suspended in Bead Solution of Ultraclean® Soil DNA isolation kit (MoBio, Carlsbad, CA). Total DNA was extracted using Ultraclean® Soil DNA isolation kit (MoBio) and the composition and abundance major groups of environmental bacteria including *Acidobacteria*, *Actinobacteria*, *Bacteroidetes*, *Firmicutes*, *Alpha-proteobacteria*, *Beta-proteobacteria*, and *Gamma -proteobacteria* determined by quantitative real-time polymerase chain reaction (qPCR) [22]. Real-time qPCR was conducted in a 20-µL reaction mixture containing 1×PCR buffer, 2 mM MgCl_2_, 1 µM dNTPs, 400 nM of both forward and reverse primers, 1×SYBR Green, 1×ROX reference dye, 1 unit of Platinum Taq DNA polymerase (Invitrogen, Grand Island, NY) and 2 µL of template DNA (20 ng/µl). Amplifications were conducted on an ABI 7300 HT sequence detection system (Applied Biosystems, Carlsbad, CA). Thermo cycling conditions for *Alpha-proteobacteria*, *Bacteroidetes*, *Firmicutes* and universal bacteria were 95°C for 5 minutes, followed by 40 cycles of 95°C for 60 seconds, 50°C for 30 seconds and 72°C for 60 seconds. For other primer sets, the PCR conditions were the same except for the annealing temperatures, which was 57°C for *Actinobacteria* and 48°C for *Beta-proteobacteria*, *Gamma-proteobacteria* and *Acidobacteria*.

Standard curves for bacterial quantification in microcosms were generated using plasmid standards with known copy numbers. Plasmid standards for primer sets specific to *Acidobacteria* and *Firmicutes* were synthesized by Integrated DNA Technologies, Inc (IDT, Coralville, Iowa) based on flanking region of 16 s rRNA gene sequences of *Terriglobus roseus* (*Acidobacteria*) and *Bacillus cereus* (*Firmicutes*) retrieved from Genbank. Plasmid standards for primer sets specific to *Actinobacteria*, *Bacteroidetes*, *Alpha-proteobacteria*, *Beta-proteobacteria*, *Gamma-proteobacteria* and universal bacteria were generated in our laboratory. Briefly, PCR amplicons for each primer sets for *Actinobacteria*, *Bacteroidetes*, *Alpha-proteobacteria*, *Beta-proteobacteria*, *Gamma-proteobacteria* or universal bacteria were run on a 1.5% agarose gel and DNA bands at expected size were excised, extracted using QIAquik gel extraction kit (Qiagen, Germantown, MD) and cloned using the TOPO TA cloning kit (Invitrogen, Carlsbad, CA). Plasmids were isolated using QIAprep spin miniprep kit (Qiagen).

The copy numbers of standard plasmids with known size were calculated based on the DNA concentration determined by NanoDrop 1000 spectrophotometer (Thermo Scientific, Pittsburg, PA) and on the assumption that the average weight of a DNA base pair (bp) is 650 Daltons. The formula for copy number calculation is: copy numbers  =  ((plasmid amounts in ng) × (6.022×10^23^))/((plasmid size in bp) ×650 ×10^9^). The concentrations of standard plasmids were adjusted to 5×10^9^ copies/µl and 10-fold serially diluted in nuclease free water (IDT). Two microliters of the serially diluted plasmid solution was utilized for qPCR. Standard curves were generated using the relationship between the cycle numbers at threshold (Ct values) and the plasmid copy numbers. The conditions of qPCR were identical to those for bacterial quantification described elsewhere in this report.

### Statistical analysis

The statistical analyses were conducted in SPSS 21 (IBM Corporation Armonk, NY, USA) and SAS 9.3 (SAS Corporation, Cary, NC, USA). Data were checked for normality and homogeneity of variances and deviations from normality were corrected through logarithm transformation. Two-way analysis of variance was used to examine the effect of leaf type and pesticide on total bacteria. Multivariate analysis of variance (MANOVA) was used to investigate the effect of leaf types and pesticides on abundance of the seven bacterial taxa. Standardized canonical coefficients were used to describe the relative contribution of each bacteria taxon to significant multivariate effects. When significant effects were detected, pairwise differences between treatment means were compared using a Tukey-Kramer adjustment. Discriminant function analysis was used to examine whether the abundance of different bacterial taxa could be used to discriminate (classify) the leaf types and pesticide treatments.

## Results

**Figure 1:**
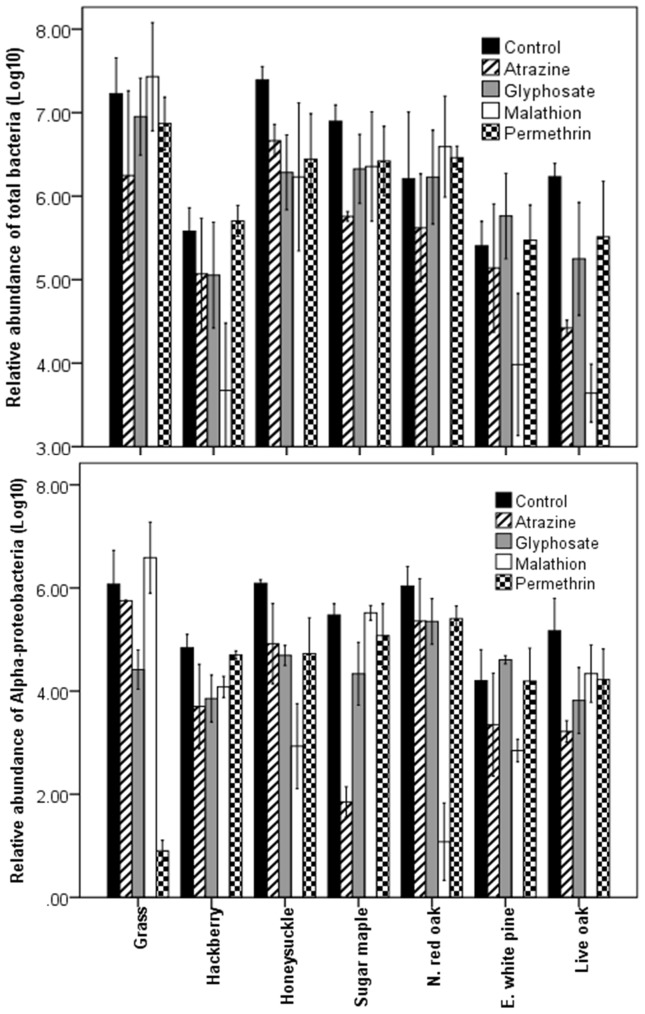
Mean number (with 95% CI) for total bacteria and *Alpha-proteobacteria* in different leaf and pesticide treatments.

A significant interaction between leaf type and pesticide was observed for total bacterial abundance (F = 30.91, df = 24, 105, *P*<0.001) implying that the effect of pesticide on bacterial abundances varied among the leaf types (Fig. 1). In grass and northern red oak, pesticide treatments had no significant effect on bacterial abundances whereas in sugar maple, honeysuckle, and live oak, bacterial abundances were significantly higher in controls compared to pesticide treatments. In eastern white pine, bacterial abundance was significantly higher in glyphosate than in malathion treatments. In hackberry, bacterial abundances were significantly higher in permethrin compared to atrazine, glyphosate, and malathion treatments but not the controls. In addition, bacteria abundances in control treatments were significantly higher compared to malathion treatments. In the absence of pesticides, bacterial abundances were highest in honeysuckle (7.4±0.05, mean ±SE), grass (7.2±0.13), and sugar maple (6.9±0.06), intermediate in live oak (6.2±0.05) and northern red oak (6.2±0.18) and lowest in hackberry (5.6±0.08) and eastern white pine (5.4±0.09).

pesticide on abundance of bacterial taxa (Table 1; Fig. 1). *Alpha-proteobacteria* (SCC = 4.97) contributed the most to significant leaf type by pesticide interactions followed by *Gamma-proteobacteria* (−1.93) and then *Firmicutes* (−1.06). In the absence of pesticides, grass, honeysuckle, and northern red oak had significantly higher abundances of *Alpha-proteobacteria* compared to live oak, hackberry, and eastern white pine but not sugar maple. In addition, sugar maple had significantly higher abundances of *Alpha-proteobacteria* compared to eastern white pine. The effect of pesticides on *Alpha-proteobacteria* varied among detritus types. In grass, malathion treatments had significantly higher abundances of *Alpha-proteobacteria* in all but control treatments. In addition, controls had significantly higher abundance of *Alpha-proteobacteria* compared to glyphosate which in turn had significantly higher abundance of *Alpha-proteobacteria* than permethrin. In hackberry, permethrin and control treatments had significantly higher abundance of *Alpha-proteobacteria* compared to the remaining treatments whereas in northern red oak, malathion had significantly lower abundance of *Alpha-proteobacteria* compared to the other treatments. In honeysuckle, abundance of *Alpha-proteobacteria* was highest in controls, intermediate in atrazine, permethrin, and glyphosate and lowest in malathion treatments. In sugar maple, abundance of *Alpha-proteobacteria* was lowest in atrazine, intermediate in glyphosate, and highest in permethrin, control, and malathion treatments. In eastern white pine, malathion and atrazine had significantly lower abundance of *Alpha-proteobacteria* compared to permethrin, control and glyphosate. In live oak, controls had significantly higher abundances of *Alpha-proteobacteria* compared to the other treatments. In addition, the abundances of *Alpha-proteobacteria* were significantly higher in permethrin and malathion treatments compared to atrazine treatment. Similar variations in abundances of *Gamma-proteobacteria* and *Firmicutes* were observed among leaf types and pesticide treatments (data not shown).

**Table 1:** Multivariate ANOVA for the effect of leaf type and pesticides on different bacterial taxa.

			Standardized canonical coefficients (SCC)						
Variable	Pillai's trace	P	*Alpha-proteobacteria*	*Beta-proteobacteria*	*Gamma-proteobacteria*	*Acidobacteria*	*Actinobacteria*	*Bacteroidetes*	*Firmicutes*
Leaf type (L)	2.83	<0.0001	−1.88	0.75	2.93	−0.42	−0.27	−0.27	2.75
Pesticide (P)	1.65	<0.0001	4.01	0.45	−1.55	0.39	0.42	0.67	−1.26
L×P	4.88	<0.0001	4.97	−0.46	−1.93	0.24	0.40	0.05	−1.06

**Figure 2:**
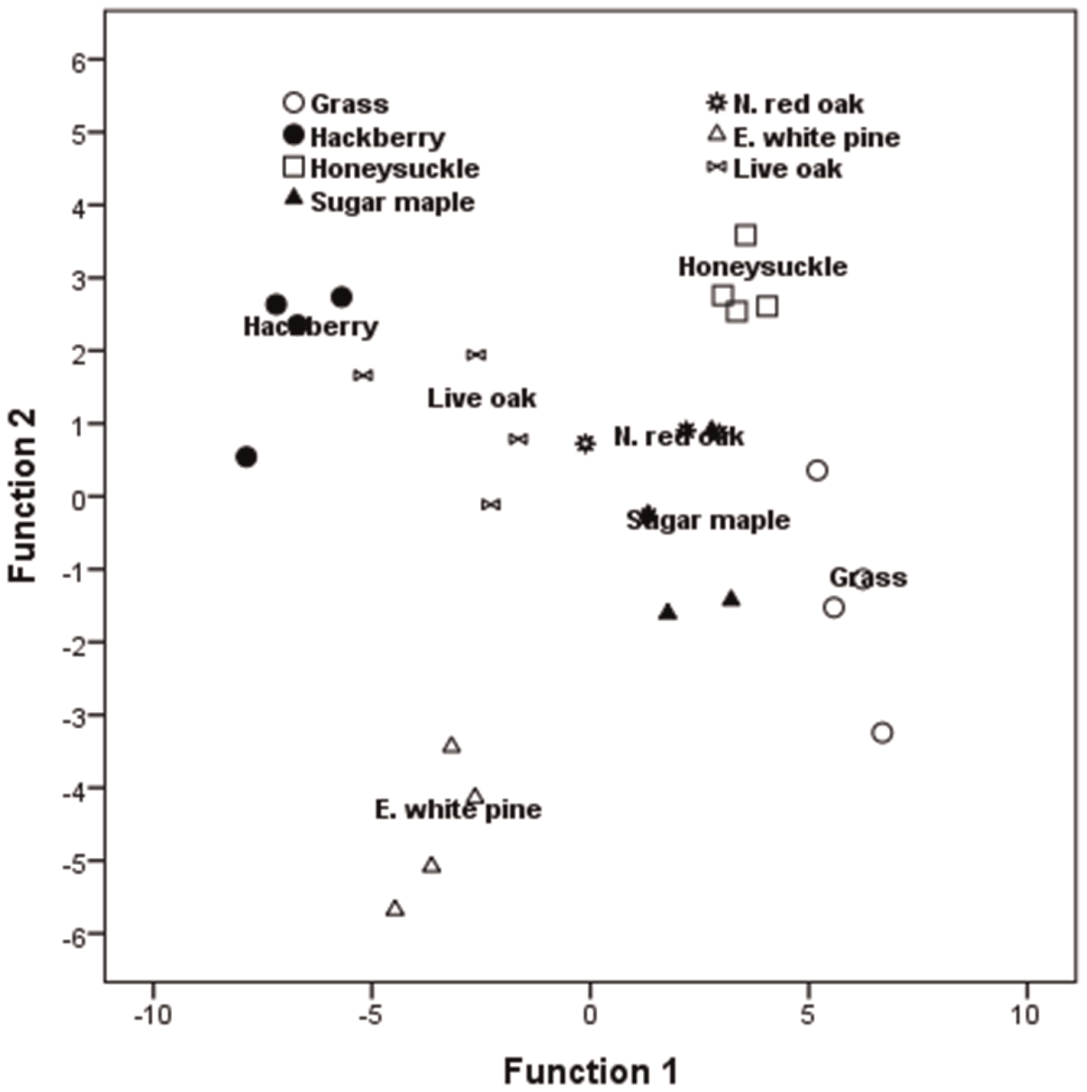
Canonical discriminant analysis showing the ordination of bacterial taxa along the first two axes and their correlations with leaf types in the absence of insecticides.

Discriminant function analysis (DFA) for the controls showed that all samples (100%) could be correctly assigned to each detritus type based on abundance of bacterial taxa. The first two discriminant functions accounted for 87% of the variation. The first discriminant function (DF) was positively correlated with *Firmicutes*, *Gamma-proteobacteria*, and *Beta-proteobacteria*, separating grass, sugar maple, honeysuckle and northern red oak (on the positive side) from hackberry, eastern white pine and live oak (Table 2, Fig. 2). The second discriminant function was positively associated with *Alpha-proteobacteria* separating hackberry, honeysuckle, live oak, and northern red oak (on the positive side) from eastern white pine, sugar maple, and grass (Table 2, Fig. 2).

**Table 2:** Discriminant function analysis results for bacterial abundance in relation to leaf type in control treatments.

	Discriminant function	
Variable	1	2
Alpha-proteobacteria	0.46	**0.51**
Beta-proteobacteria	**0.49**	0.17
Gamma-proteobacteria	**0.46**	0.19
Acidobacteria	0.30	0.40
Actinobacteria	0.19	0.07
Bacteroidetes	0.31	0.19
Firmicutes	**0.78**	0.06
Summary statistics		
Wilk's lambda	0.001	0.01
Chi square	159.0	95.1
*P* value	<0.0001	<0.0001
Eigen values (%)	66.7	20.2
Canonical correlation	0.98	0.94

Analysis was conducted using log transformed values.

**Figure 3:**
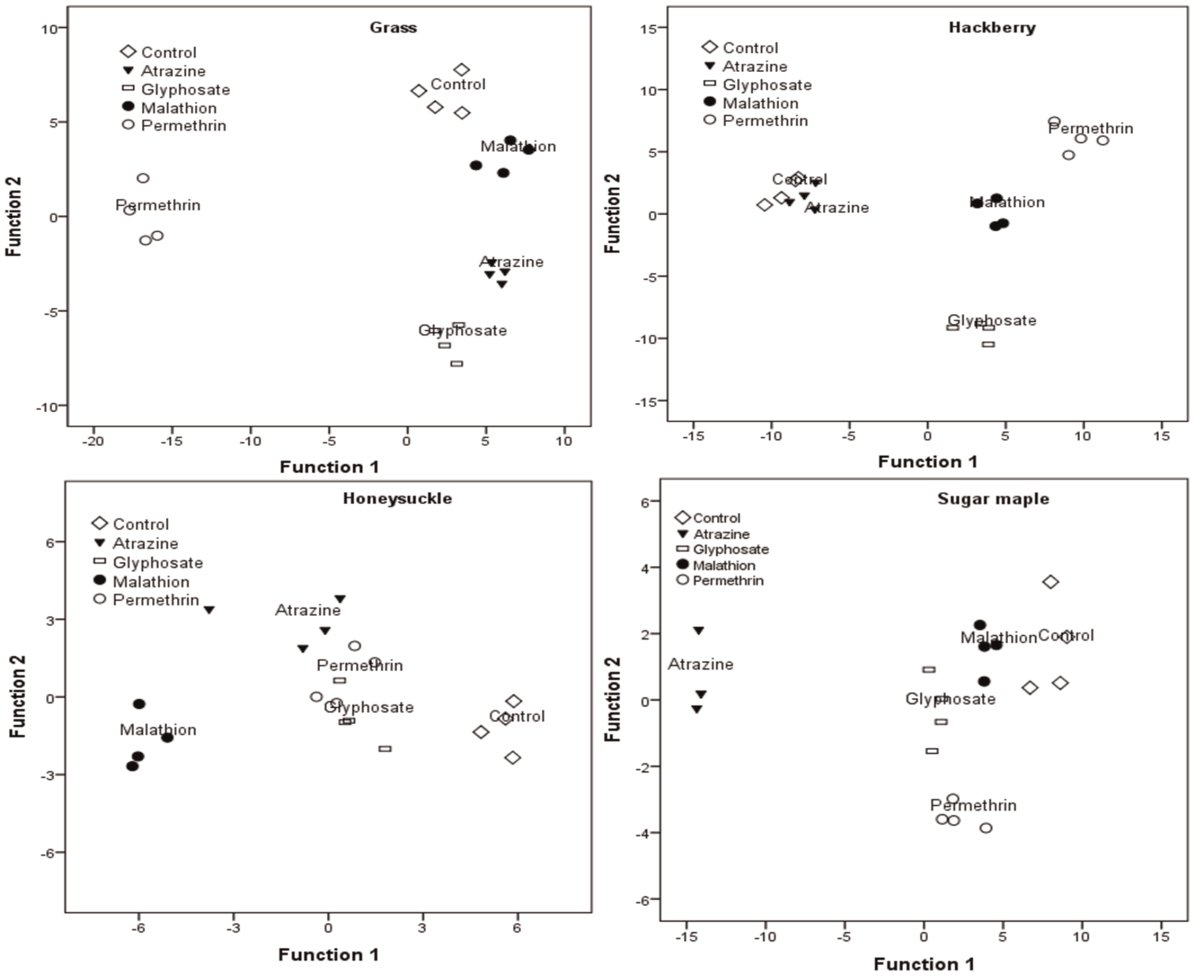
Canonical discriminant analysis showing the ordination of bacterial taxa along the first two axes and their correlations with pesticide treatments in grass, hackberry, honeysuckle and sugar maple. These are rapid decaying leaf types.

**Figure 4:**
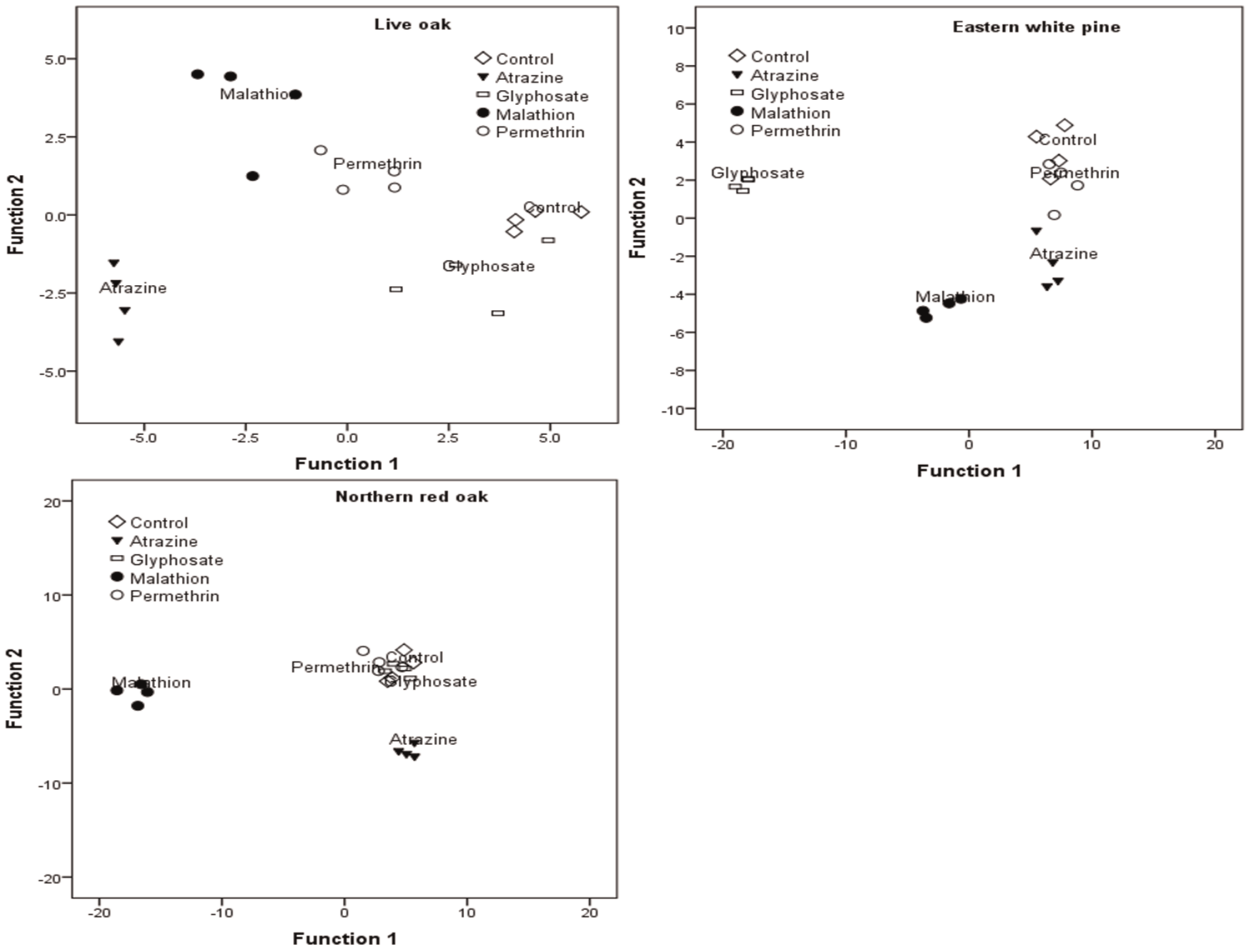
Canonical discriminant analysis showing the ordination of bacterial taxa along the first two axes and their correlations with pesticide treatments in live oak, eastern white pine and northern red oak. These are slow decaying leaf types.

MANOVA revealed a significant interaction between leaf type and In order to determine how pesticide treatments differed with respect to their effect on bacterial taxa within leaf types, we conducted separate discriminant function analysis for each leaf type. The first two discriminant functions were statistically significant for each leaf type. However, some bacteria taxa loaded significantly in both DFs and were interpreted for the function on which they loaded the highest (Table 3, Fig. 3 and 4). Ninety five percent of samples in honeysuckle and northern red oak were correctly assigned to pesticide treatments while all the samples (100%) in the remaining leaf types were correctly assigned to pesticide treatments.

**Table 3:** Discriminant function analysis results for bacterial abundance in relation to pesticide treatments in different leaf types.

		Function	
		1	2
Grass	Alpha-proteobacteria	**0.78**	0.44
	Beta-proteobacteria	0.09	**0.52**
	Acidobateria	0.13	**0.57**
	P value	<0.001	<0.001
	Eigen value	76.5	21.7
Hackberry	Bacteroidetes	**−0.51**	0.13
	Gamma-proteobacteria	−0.075	**0.54**
	P value	<0.001	<0.001
	Eigen value	62.8	31.1
Honeysuckle	Acidobacteria	**0.42**	−0.14
	Alpha-proteobacteria	**0.70**	0.28
	Beta-proteobacteria	**0.51**	−0.24
	Gamma-proteobacteria	0.35	**0.40**
	P value	<0.001	0.001
	Eigen value	76.6	16.1
Sugar maple	Acidobacteria	0.20	**0.54**
	Alpha-proteobacteria	**0.63**	0.17
	Beta-proteobacteria	0.11	**0.49**
	Firmicutes	0.08	**−0.32**
	P value	<0.001	0.001
	Eigen value	90.7	5.2
Live oak	Alpha-proteobacteria	0.35	**0.44**
	Bacteroidetes	**0.66**	0.12
	Beta-proteobacteria	**0.55**	0.13
	Firmicutes	0.18	**0.39**
	P value	<0.001	<0.001
	Eigen value	61.8	22.3
Northern red oak	Alpha-proteobacteria	**0.58**	0.16
	Beta-proteobacteria	0.07	**0.94**
	P value	<0.001	<0.001
	Eigen value	84.1	13.8
Eastern white pine	Actinobacteria	0.32	**0.50**
	Alpha-proteobacteria	−0.05	**0.51**
	Bacteroidetes	0.04	**0.33**
	Firmicutes	**0.74**	−0.11
	P value	<0.001	<0.001
	Eigen value	88.9	8.7

Only bacteria taxa with significant DF functions are presented.

1. Grass: *Alpha-proteobacteria* loaded highly on the positive side of DF1 separating permethrin (on the negative side) from the other treatments. In contrast, *Beta-proteobacteria* and *Acidobacteria* loaded highly on the positive side of DF2, separating control and malathion treatments (positive side) from atrazine and glyphosate treatments.
2. Hackberry: *Bacteroidetes* loaded highly on the negative side of DF1, separating control and atrazine treatments (on the negative side) from the remaining treatments. DF2 had high loads for *Gamma-proteobacteria* on the positive side separating glyphosate (negative side) from the other treatments.
3. Honeysuckle: DF1 had high loads for *Acidobacteria*, *Alpha*- and *Beta-proteobacteria* on the positive side, separating control, glyphosate and permethrin treatments (positive side) from atrazine and malathion treatments (negative side). DF2 was positively associated with *Gamma-proteobacteria* separating atrazine and permethrin (positive side) from the remaining treatments.
4. Sugar maple: *Alpha-proteobacteria* was positively correlated with DF1, separating control, malathion, and permethrin on the positive side from atrazine on the negative side. DF2 was highly correlated with *Firmicutes* on the negative side and *Beta-proteobacteria* and *Acidobacteria* on the positive side. This separated permethrin (on the negative side) from the other treatments.
5. Live oak: DF1 was positively correlated with *Bacteroidetes* and *Beta-proteobacteria* separating control and glyphosate on the positive side from atrazine and malathion on the negative side. DF2 was positively correlated with *Alpha-proteobacteria* and *Firmicutes* separating malathion and permethrin on the positive side from the other treatments.
6. Northern red oak: DF1 was positively correlated with *Alpha-proteobacteria* while DF2 was positively correlated with *Beta-proteobacteria*. DF1 separated malathion (on the negative side) from the other treatments, while DF2 separated atrazine from control, permethrin, and glyphosate treatments.
7. Eastern white pine: *Firmicutes* were positively correlated with DF1, separating glyphosate and malathion on the negative side from the other treatments which loaded highly on the positive side. DF2 was a linear combination of high loads for *Alpha-proteobacteria*, *Actinobacteria* and *Bacteroidetes* on the positive side separating atrazine and malathion (on the negative side) from the other treatments which tended to load more on the positive side.

## Discussion

The quantities of both total bacteria and specific bacteria phyla and sub-phyla varied significantly among leaf types. Previous studies reported that rapidly decaying detritus types support higher quantities of total bacteria compared to slow decaying detritus [10]. Our data were consistent with these findings given that three of four rapidly decomposing leaf types had the highest quantities of total bacteria while eastern white pine, which has the slowest decay rate among the 7 leaf types, had the lowest quantities of total bacteria. However, the fact that the quantities of total bacteria in hackberry (a rapidly decaying leaf type) were identical to those of eastern white pine suggests that decay rate is not a universal indicator of microbial abundance in leaf detritus. Similarly, discriminant function analysis revealed a tendency for rapidly decaying leaf types to be significantly associated with *Beta-proteobacteria*, *Gamma-proteobacteria* and *Firmicutes* but this trend was not universal given that the northern red oak (slow decaying) grouped together with rapidly decaying leaf types while hackberry (rapid decaying) grouped together with the slowly decaying leaf types. Although we did not quantify mosquito growth and development in response to detritus type, we believe that low microbial abundance in some rapidly decaying leaf litter such as hackberry may explain why most but not all rapidly decaying leaf detritus types promote mosquito growth and survival and eliminate interspecific competition among larvae of container-dwelling mosquitoes [10]–[12]. Further studies are needed on this topic.

Pesticide treatments had neutral or negative effects on abundance of total bacteria and on major bacterial phyla and sub-phyla. These effects varied among leaf types, where a pesticide had toxic effects on bacterial communities in some leaf types and neutral effects on others. For example, all pesticides had negative effects on abundance of total bacteria in sugar maple, honeysuckle, and live oak infusions and neutral effects in grass and northern red oak infusions. Similarly, all pesticides had negative effects on abundance of *Alpha-proteobacteria* in honeysuckle and live oak infusions, whereas in grass, hackberry, northern red oak and eastern white pine, some pesticides had negative effects while others had neutral effects. These findings suggest that interactions between leaf types, pesticides and microbes can be highly complex and difficult to predict. The observed neutral effects of pesticides could be attributed to the replacement of pesticide-sensitive bacteria by pesticide-tolerant bacteria. Some microbes are capable of using pesticides as a source of energy and may occupy the vacant ecological niche left by the pesticide-sensitive bacteria [23]–[25]. In fact, an increase in the resistant portion of the microbial community is a commonly reported effect of chemical contaminants in aquatic environments [16], [17], [26], [27]. These microbes can mask the effect of pesticides on microbial communities by compensating for microbial biomass, bacterial activity and respiratory deficits associated with loss of pesticide-sensitive microbes [17], [28], [29].

Our results revealed pesticide-induced shifts in bacterial community structure in all leaf types, suggesting that certain groups of bacteria benefitted from low concentrations of pesticides. Higher abundance of some bacteria in pesticide treatments may have resulted from their ability to use pesticides as a carbon source or by elimination of pesticide-sensitive species thereby releasing pesticide-tolerant bacteria from competition. Pesticide-mediated competitive-release has been reported in microbial communities [24], [30] and some members of *Proteobacteria*, *Actinobacteria*, *Firmicutes*, *Bacteroidetes* and *Acidobacteria* are important degraders of pesticides and are therefore expected to form significant populations in pesticide-contaminated sites [31]–[36]. Pesticide-induced changes in microbial community structure have been reported from a number of fungicides, herbicides and insecticides in both terrestrial and aquatic environments [16], [29], [37], [38]. These shifts may lead to succession in microbial communities that may affect ecological processes in mosquito aquatic habitats and other fresh water ecosystems.

Like other ecological studies, this study had several limitations. First, we quantified the bacterial component of microbial assemblages but not the protozoan and the fungal components. However, although larvae of container mosquitoes consume many types of protozoans and fungi, bacterial communities are known to be their primary food source [8], [39]. Second, we quantified the bacterial communities in the water column only yet mosquito larvae also consume leaf-surface associated microbes [39]. Future studies should examine the impact of anthropogenic disturbances on microbial assemblages in both water column and leaf surfaces. Finally, the use of conserved regions of 16S rDNA to detect and quantify bacteria in complex communities is not without limitations. DNA extraction bias may alter the estimated abundances of certain groups, heterogeneity in ribosomal operon number may affect relative estimates of group abundances and the tested qPCR assays do not necessarily amplify rRNA genes belonging to all members of each target group [40], [41]. These drawbacks may lead to underestimation of the true abundance of the target bacterial communities. However, we believe our results are a good starting point to the understanding of microbial communities in mosquito larval habitats and the impact of anthropogenic chemical contamination on these communities.

In summary, we examined the impact of leaf type and exposure to pesticides on microbial community structure at the coarsest level of taxonomic resolution. We found significant differences in bacterial abundances among different leaf types. Further, pesticide treatments induced shifts in bacterial community structure but these effects varied among leaf types. Bacterial communities associated with decaying leaf litter constitute a major food base for mosquito larvae [8], [9], [39] and produce chemical cues that act as oviposition attractants and stimulants for gravid mosquitoes [5]–[7]. Therefore, further studies are required to directly link changes in bacterial abundance and community structure associated with detritus type and pesticide exposure to mosquito production and thereby the risk of mosquito-borne diseases.
